# Interleukin-26 is overexpressed in human sepsis and contributes to inflammation, organ injury, and mortality in murine sepsis

**DOI:** 10.1186/s13054-019-2574-7

**Published:** 2019-08-29

**Authors:** Hongmei Tu, Xiaofei Lai, Jiaxi Li, Lili Huang, Yi Liu, Ju Cao

**Affiliations:** 1 Department of Laboratory Medicine, The First Affiliated Hospital of Chongqing Medical University, No. 1 Youyi Road, Yuzhong District, Chongqing, 400016 China; 2 Department of Intensive care unit, The Second Affiliated Hospital of Chongqing Medical University, Chongqing, China

**Keywords:** Sepsis, Inflammation, IL-26, Neutrophils, Mortality

## Abstract

**Background:**

Sepsis is a serious syndrome that is caused by an unbalanced host inflammatory response to an infection. The cytokine network plays a pivotal role in the orchestration of inflammatory response during sepsis. IL-26 is an emerging proinflammatory member of the IL-10 cytokine family with multifaceted actions in inflammatory disorders. However, its role in the pathogenesis of sepsis remains unknown.

**Methods:**

Serum IL-26 level was measured and analyzed in 52 septic patients sampled on the day of intensive care unit (ICU) admission, 18 non-septic ICU patient controls, and 30 healthy volunteers. In addition, the effects of recombinant human IL-26 on host inflammatory response in cecal ligation and puncture (CLP)-induced polymicrobial sepsis were determined.

**Results:**

On the day of ICU admission, the patients with sepsis showed a significant increase in serum IL-26 levels compared with ICU patient controls and healthy volunteers, and the serum IL-26 levels were related to the severity of sepsis. Nonsurvivors of septic patients displayed significantly higher serum IL-26 levels compared with survivors. A high serum IL-26 level on ICU admission was associated with 28-day mortality, and IL-26 was found to be an independent predictor of 28-day mortality in septic patients by logistic regression analysis. Furthermore, administration of recombinant human IL-26 increased lethality in CLP-induced polymicrobial sepsis. Despite a lower bacterial load, septic mice treated with recombinant IL-26 had higher concentrations of IL-1β, IL-4, IL-6, IL-10, IL-17A, TNF-α, CXCL1, and CCL2 in peritoneal lavage fluid and blood and demonstrated more severe multiple organ injury (including lung, liver and kidney) as indicated by clinical chemistry and histopathology. Furthermore, septic mice treated with recombinant human IL-26 showed an increased neutrophil recruitment to the peritoneal cavity.

**Conclusions:**

Septic patients had elevated serum IL-26 levels, which may correlate with disease severity and mortality. In experimental sepsis, we demonstrated a previously unrecognized role of IL-26 in increasing lethality despite promoting antibacterial host responses.

**Electronic supplementary material:**

The online version of this article (10.1186/s13054-019-2574-7) contains supplementary material, which is available to authorized users.

## Background

Sepsis is a common disorder that is associated with unacceptably high mortality and, for many of those who survive, long-term morbidity. In 2016, a new definition of sepsis (Sepsis-3) was developed [1]. Sepsis is now defined as a life-threatening organ dysfunction that is caused by a dysregulated host response to infection [1]. In 2017, the World Health Assembly and WHO made sepsis a global health priority [2]. In sepsis, the host immune response has departed from homeostasis in two opposite directions by showing signs of both excessive inflammation and immune suppression, the extent of which varies between individuals [3]. Knowledge of the complex immunopathological mechanisms regulating the inflammatory host response during sepsis is essential for precision medicine in sepsis.

Interleukin-26 (IL-26) is an emerging member of IL-10 family cytokines [4]. IL-26 is highly conserved in vertebrate species but absent in mice [5]. IL-26 can be produced by primary T cells, NK cells, and T cell clones following stimulation with specific antigen or mitogenic lectins. IL-26 signals via the heterodimeric IL-20R1/IL-10R2 receptor and induces Janus kinase-signal transducer and activator of transcription (JAK-STAT) activation, resulting in STAT1 and STAT3 phosphorylation [5]. IL-26 expression was upregulated in the patients with rheumatoid arthritis, hepatitis C virus (HCV) infection [6], inflammatory bowel disease [7], or asthma [8]. Microarray analysis of *Mycobacterium tuberculosis*-infected monocytes has revealed IL-26 as a new candidate gene for tuberculosis susceptibility [9]. IL-26 thereby appears as a new player in the inflammatory and immune responses associated with autoimmune and infectious diseases.

In this study, we aimed to determine the extent of systemic IL-26 expression in patients with sepsis and to investigate the relationship of IL-26 to disease severity and survival in human sepsis. Although IL-26 expression is absent in mice, mice do express both receptor chains that are necessary to form IL-26 receptor complexes (IL-20R1 and IL-10R2) [5]. Therefore, it is certainly possible that human IL-26 protein can bind and signal via murine IL-26 receptor complexes to mediate its potential biological functions. In addition, we examined the potential role of IL-26 in host immune responses to murine sepsis by administration of recombinant human IL-26 protein. Overall, our data demonstrated that IL-26 contributed to inflammatory response, organ injury, and mortality in murine sepsis despite enhancing the host’s ability to fight pathogens.

## Methods

### Human patients and controls

Fifty-two adult septic patients who met the clinical criteria for sepsis-3 [1] were enrolled on admission to the intensive care unit (ICU) of The Second Affiliated Hospital of Chongqing Medical University at the day sepsis was diagnosed. Patients with pregnancy or breast-feeding, malignancy, organ transplantation, chronic viral infections (hepatitis, HIV), liver cirrhosis, chronic kidney insufficiency, autoimmune diseases, and the use of immunosuppressive medication in the past 8 weeks were excluded from the study. Eighteen non-septic patients but in critical conditions (poly-trauma, cerebral trauma or burns) were recruited as patient controls. The clinical data, including Sequential Organ Failure Assessment (SOFA) score, the counts of white blood cells (WBC), the levels of procalcitonin (PCT) and C-reaction protein (CRP), microbial culture results, the length of ICU stay, or mortality during the 28-day study period, were recorded. There was no difference in end-stage renal failure between septic patients and non-septic patients. In addition, 30 age- and sex-matched healthy adult control samples were obtained from healthy donors with no medical problems in the medical examination center of The Second Affiliated Hospital of Chongqing Medical University. This protocol was approved by the Clinical Research Ethics Committee of Chongqing Medical University, and informed consent was obtained from all participants according to the Declaration of Helsinki.

### Sepsis model

Pathogen-free 6–8-week-old female C57BL/6 mice were obtained from and raised at Chongqing Medical University. Polymicrobial sepsis was provoked by cecal ligation and puncture (CLP) as described in our previous studies [10, 11]. C57BL/6 mice were anesthetized with a mixture of xylazine (4.5 mg/kg) and ketamine (90 mg/kg) intraperitoneally (i.p.). CLP was performed by making a midline incision ~ 2.5 cm in length to expose the cecum. A 3-0 silk ligature was placed at the base of the cecum without causing bowel obstruction. The cecum was then punctured once with a 22-gauge needle. The cecum was then placed back in the peritoneal cavity, and the incision was closed with surgical staples. Sham-operated (control) animals underwent identical laparatomy; the cecum was exposed but not ligated or punctured and was then replaced in the peritoneal cavity. Mice received saline (5 ml per 100 g body weight) subcutaneously for resuscitation, and no antibiotic treatment was used. Survival was monitored twice daily for 14 days. The animal experiments were approved by the local Animal Care and Use Committee.

### In vivo administration of recombinant human IL-26

Female 6- to 8-week-old C57BL/6 mice were purchased from the Animal Center of Chongqing Medical University and allowed to acclimatize at a specific-pathogen-free facility at Chongqing Medical University for 1 week. Recombinant human IL-26 protein (R&D systems) was injected immediately after CLP. PBS was delivered in a similar fashion as control vehicle.

### Cytokine and chemokine measurement

The concentration of human IL-26 was determined by human IL-26 ELISA kit (LifeSpan Biosciences). The levels of IL-1β, TNF-α, IL-4, IL-6, IL-10, IL-17A, CXCL1, and CCL2 were determined with commercially available ELISA kits (Biolegend) according to the manufacturer’s instructions. For measurement of cytokines and chemokines in peritoneal lavage fluid (PLF), 5 ml of cold PBS solution was injected into the peritoneal cavity. PLF was collected for complete analysis of chemokines/cytokines by ELISA.

### Serum biochemistry

Blood was collected from mice in tubes with heparin after cardiac puncture, centrifuged (10 min, 1600×*g*). Alanine aminotransferase (ALT), aspartate transaminase (AST), lactate dehydrogenase (LDH), and creatinine were measured according to the protocols of the International Federation of Clinical Chemistry, by spectrophotometric analysis (modular DP; Roche-Hitachi, Echevarne Laboratories).

### Determination of bacterial colony-forming unit (CFU) in the CLP model

Mice were killed at the indicated times following CLP. The peritoneal cavities were washed with 2 ml sterile PBS, and the lavage fluids were harvested under sterile conditions. The spleens were isolated, and 10 mg of each tissue was homogenized in 700 μl of PBS. A total of 10 μl of each dilution was asceptically plated on tryptose soy agar blood agar plates (Difco, Detroit, MI) and incubated overnight at 37 °C, after which the number of colonies was counted.

### Histopathology

Mice subjected to sham or CLP were killed at the indicated times after surgery, after which their lungs, livers, kidneys, and spleens were fixed in 4% formalin and embedded in paraffin. Four-micrometer sections were stained with hematoxylin and eosin (H&E) and analyzed by a pathologist blinded for groups using a BH2 Olympus microscope (Olympus). To score lung inflammation and damage, the entire lung surface was analyzed with respect to the following parameters: bronchitis, edema, interstitial inflammation, intra-alveolar inflammation, pleuritis, endothelialitis, and percentage of the lung surface demonstrating confluent inflammatory infiltrate. Each parameter was graded from 0 (absent) to 4 (severe). Livers, kidneys, and spleens were scored according to the following parameters: number of thrombi, number of (micro) abscesses, presence and degree of inflammation, and presence and degree of necrosis. Each parameter was graded from 0 (absent) to 3 (severe). The total pathology scores for the lungs, livers, kidneys, and spleens were expressed as the sum of the score for all parameters.

### Differential cell counts in the peritoneum

Peritoneal lavage was performed with 5 mL of PBS containing 1 mM ethylenediaminetetraacetic acid. Peritoneal cell suspension was pelleted and resuspended. Cell viability was determined using Trypan blue exclusion assay, and cell numbers were counted with a hematology analyzer. Cytospin slides were prepared and stained with a Wright-Giemsa stain.

### Macrophage/neutrophil phagocytosis assays

For isolation of peritoneal macrophages, mice were injected with 5 ml PBS. Macrophages were isolated from peritoneal lavage by plastic adherence. For isolation of granulocytes, mice were injected intraperitoneally with 1.5 ml sterile thioglycollate (3%). Elicited cells were harvested 4 h later by peritoneal lavage with 5 ml of cold PBS, and neutrophils were further purified from peritoneal lavage by magnetic cell sorting (Miltenyi Biotec). FITC-labeled *E. coli* were prepared by incubation with 0.5 mg/ml FITC (Sigma) for 20 min at 37 °C. Peritoneal macrophages (1 × 10^5^ cells) or neutrophils (1 × 10^6^ cells) were incubated with FITC-labeled bacteria at a multiplicity of infection of 100 for 30 min at 37 °C. After washing steps, cell nuclei were stained with DAPI (Invitrogen), followed by visualization using confocal laser scanning microscopy (LSM 510, Zeiss). The ratio of engulfed bacteria (as determined by overlay of green bacteria) was quantified by an independent researcher from 300 counted cells per well. In some experiments, peritoneal macrophages and neutrophils were pretreated with recombinant human IL-26 (100 ng/ml, R&D systems) before infection by FITC-labeled *E. coli*.

### Macrophage/neutrophil bacterial killing assays

Peritoneal macrophages (1 × 10^5^ cells) were infected with live *E. coli* (multiplicity of infection, 10) at 37 °C for 1 h, and they were washed with buffer containing tobramycin (100 μg/ml) to remove extracellular bacteria and were lysed with lysis buffer (Promega). Live intracellular bacteria were quantified by culture of lysates for determination of intracellular killing (*t* = 2 h). Killing was calculated from the percentage of colonies present at *t* = 2 h as compared to *t* = 0 h, as follows: 100 − [number of CFUs *t* = 2 h/number of CFUs *t* = 0 h]. In another experiment, peritoneal neutrophils (1 × 10^6^ cells) were infected with *E. coli* at an MOI ratio of 1:100 at 37 °C for 30 min, and cells were resuspended in a medium containing 100 μg/mL tobramycin to remove extracellular bacteria and then lysed in PBS containing 0.1% Triton 100, and samples were then incubated for 1 additional hour (*t* = 1 h) to assess bacterial killing as described above. In some experiments, peritoneal macrophages and neutrophils were pretreated with recombinant human IL-26 (100 ng/ml, R&D systems) before infection by live *E. coli*.

### Statistical analysis

Human data were expressed as scatter dot plots with medians. Mice data were expressed as box-and-whisker plots showing the smallest observation, lower quartile, median, upper quartile, and largest observation or as medians with interquartile ranges. Cell data were expressed as mean ± standard deviation (SD). The Mann–Whitney *U* test or Kruskal-Wallis test followed by Dunn’s multiple comparisons post test was performed for data of non-normal distribution. Student’s *t* test was for data of normal distribution. Non-parametric Spearman’s rank correlation coefficient was used to test correlations between two parameters. To determine the discriminative power of IL-26 for 28-day mortality, receiver-operating characteristic (ROC) curves were constructed and the area under the curve (AUC) was determined with its 95% confidence interval (CI). For comparisons of AUCs, the *Z*-test formula was applied. Binary logistic regression analysis was applied to determine the independent predictors of 28-day mortality. For murine survival studies, Kaplan–Meier analyses followed by log-rank tests were performed. All analyses were done using GraphPad Prism version 5.01 (GraphPad Software, San Diego, CA). *p* values less than 0.05 were considered statistically significant.

## Results

### Sepsis resulted in elevated serum IL-26 levels

**Fig. 1:**
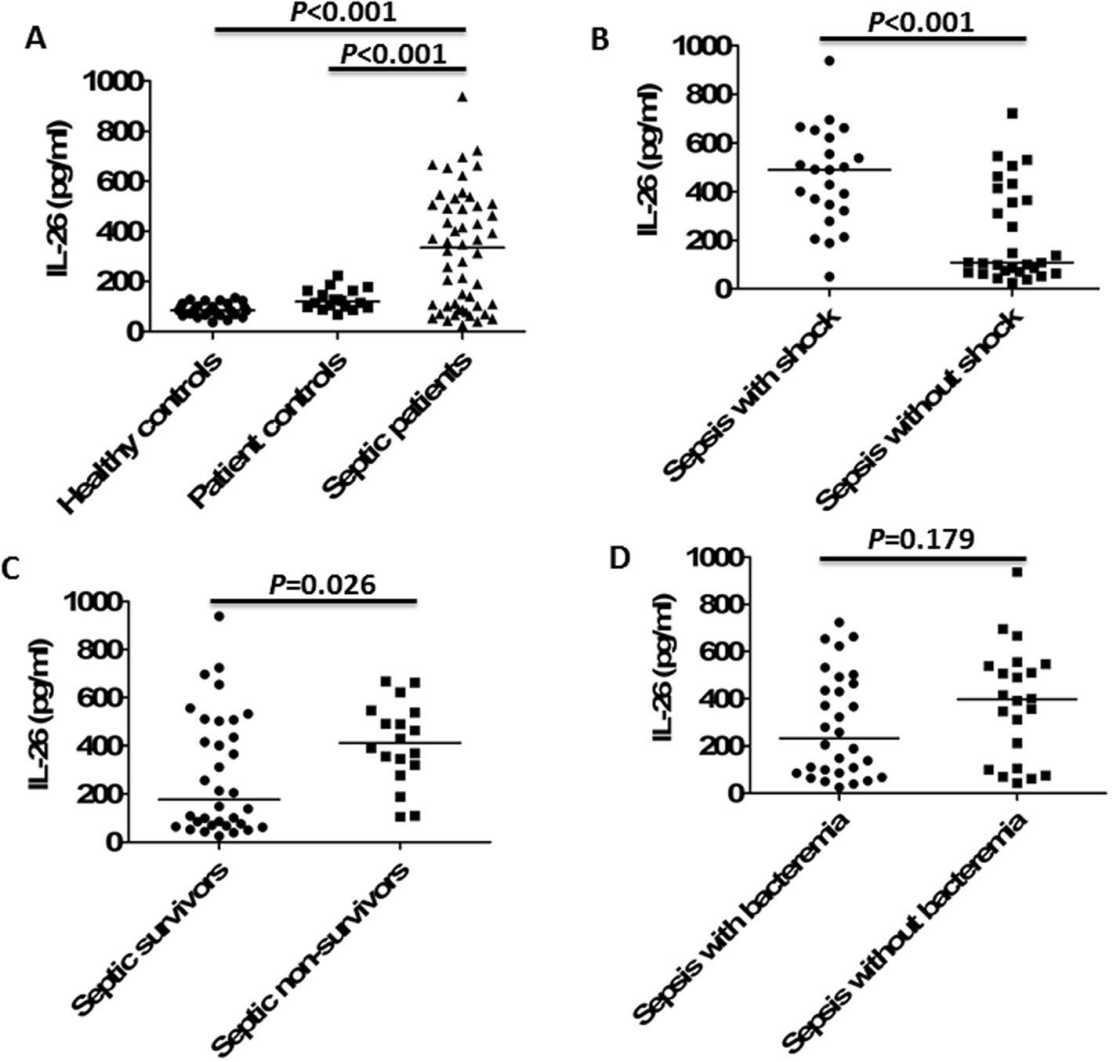
Serum IL-26 levels at admission were elevated in the patients with sepsis. **a** IL-26 concentrations were measured by ELISA in serum samples collected from 52 patients with sepsis, 18 non-septic ICU controls, and 30 healthy control subjects. **b** IL-26 concentrations in serum samples collected from septic shock patients and patients without shock. **c** IL-26 concentrations in serum samples collected from survivors and nonsurvivors in the patients with sepsis. **d** IL-26 concentrations in serum samples collected from septic patients with bacteremia and patients without bacteremia. Non-parametric Mann–Whitney *U* test or Kruskal-Wallis test followed by Dunn’s multiple comparisons post test was used to compare results between groups. Horizontal bars represent median values, and dots represent individual participants

The demographic and clinical characteristics of patients with sepsis, patient and healthy controls were presented in Additional file 4: Table S1. The 28-day mortality was 34.6% in these septic patients. Non-survivors in septic patients had significantly higher SOFA scores and lactate levels when compared to survivors on ICU admission (Additional file 5: Table S2). In the serum of 52 septic patients on the day of ICU admission, IL-26 levels were significantly higher than patient controls and healthy individuals (Fig. 1a). Serum IL-26 concentrations on ICU admission were significantly increased in septic shock patients compared to septic patients without shock (Fig. 1b), and non-surviving patients with sepsis showed significantly higher IL-26 levels compared to survivors with sepsis (Fig. 1c). Furthermore, septic patients with bacteremia had lower circulating IL-26 levels when compared to non-bacteremic patients. However, it did not reach statistical difference (Fig. 1d).

### The clinical role of IL-26 in diagnosing sepsis and predicting 28-day mortality

**Fig. 2:**
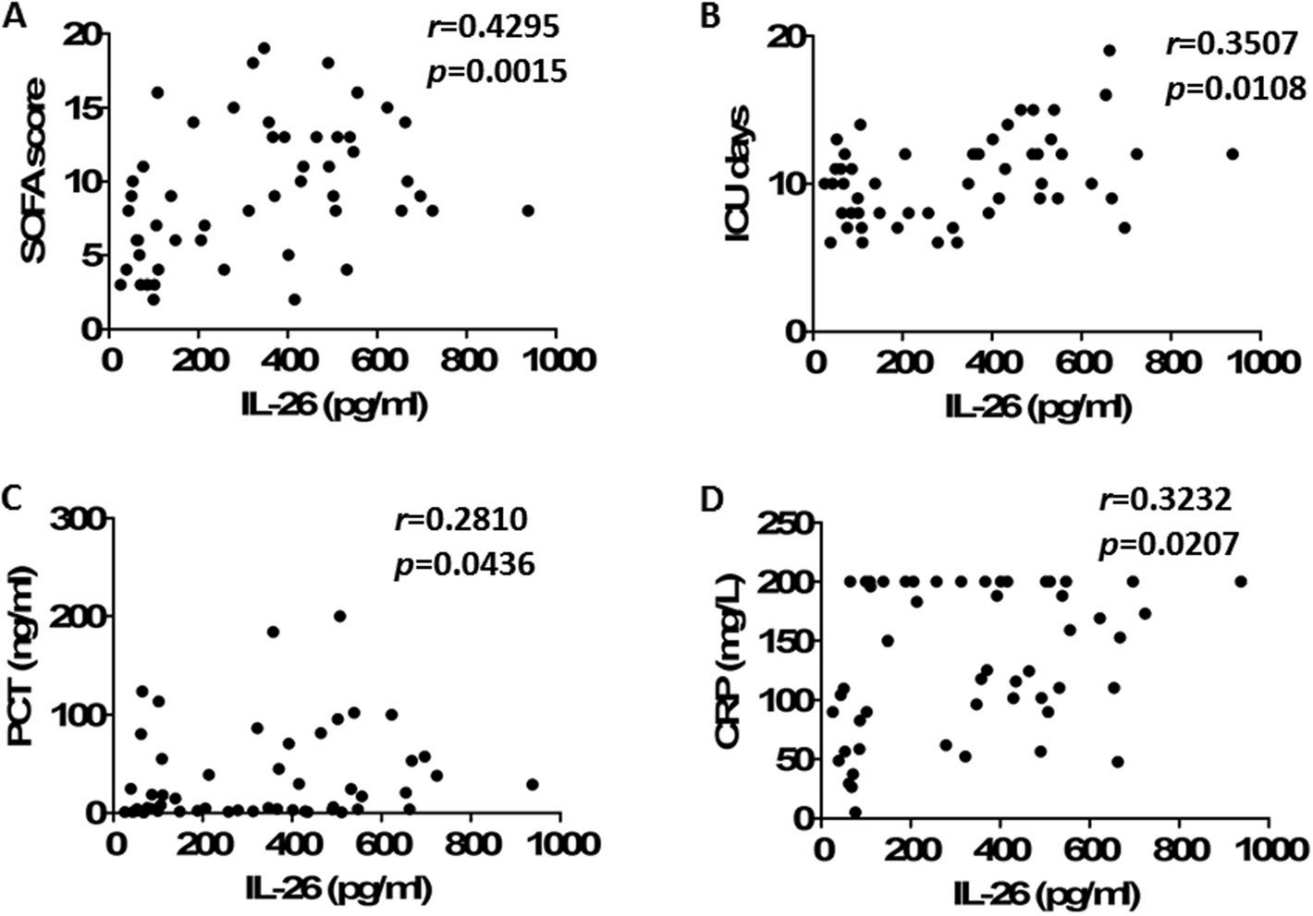
Serum IL-26 levels at admission correlated with disease severity in septic patients. **a** Correlation of IL-26 levels with Sequential Organ Failure Assessment (SOFA) scores in the patients with sepsis. **b** Correlation of IL-26 levels with the length of ICU stay in the patients with sepsis. **c** Correlation of IL-26 levels with procalcitonin (PCT) levels in the patients with sepsis. **d** Correlation of IL-26 levels with C-reactive protein (CRP) levels in the patients with sepsis. Spearman’s correlation coefficient was used to evaluate the correlation between concentrations of IL-26 and SOFA scores, the length of ICU stay, PCT, or CRP levels. Horizontal bars represent median values, and dots represent individual participants

Higher serum IL-26 levels were related to higher SOFA scores in the patients with sepsis on day of ICU admission (Fig. 2a). Moreover, serum IL-26 levels increased with the length of ICU stay (Fig. 2b). Additionally, there was a significant correlation between IL-26 and PCT (Fig. 2c), or CRP (Fig. 2d) levels on ICU admission.

**Fig. 3:**
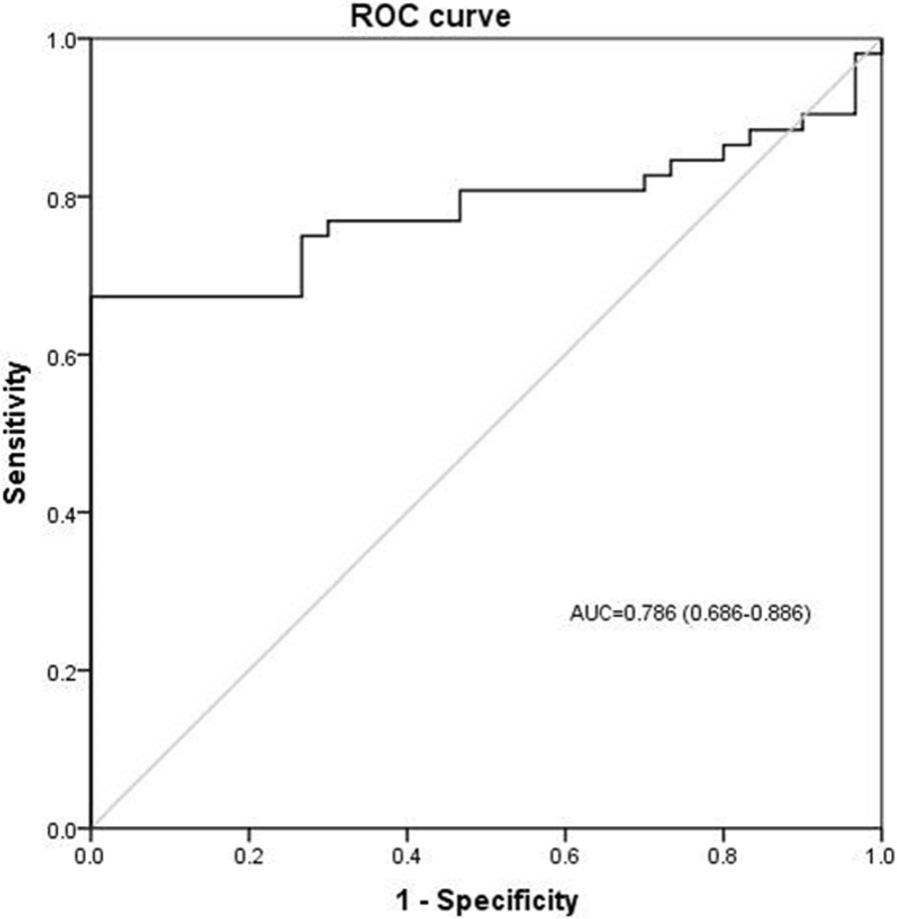
Receiver operating characteristic curve (ROC) of IL-26 for diagnosis of sepsis. Areas under the ROC curve for IL-26, 0.786 (*p* = 0.01)

**Fig. 4:**
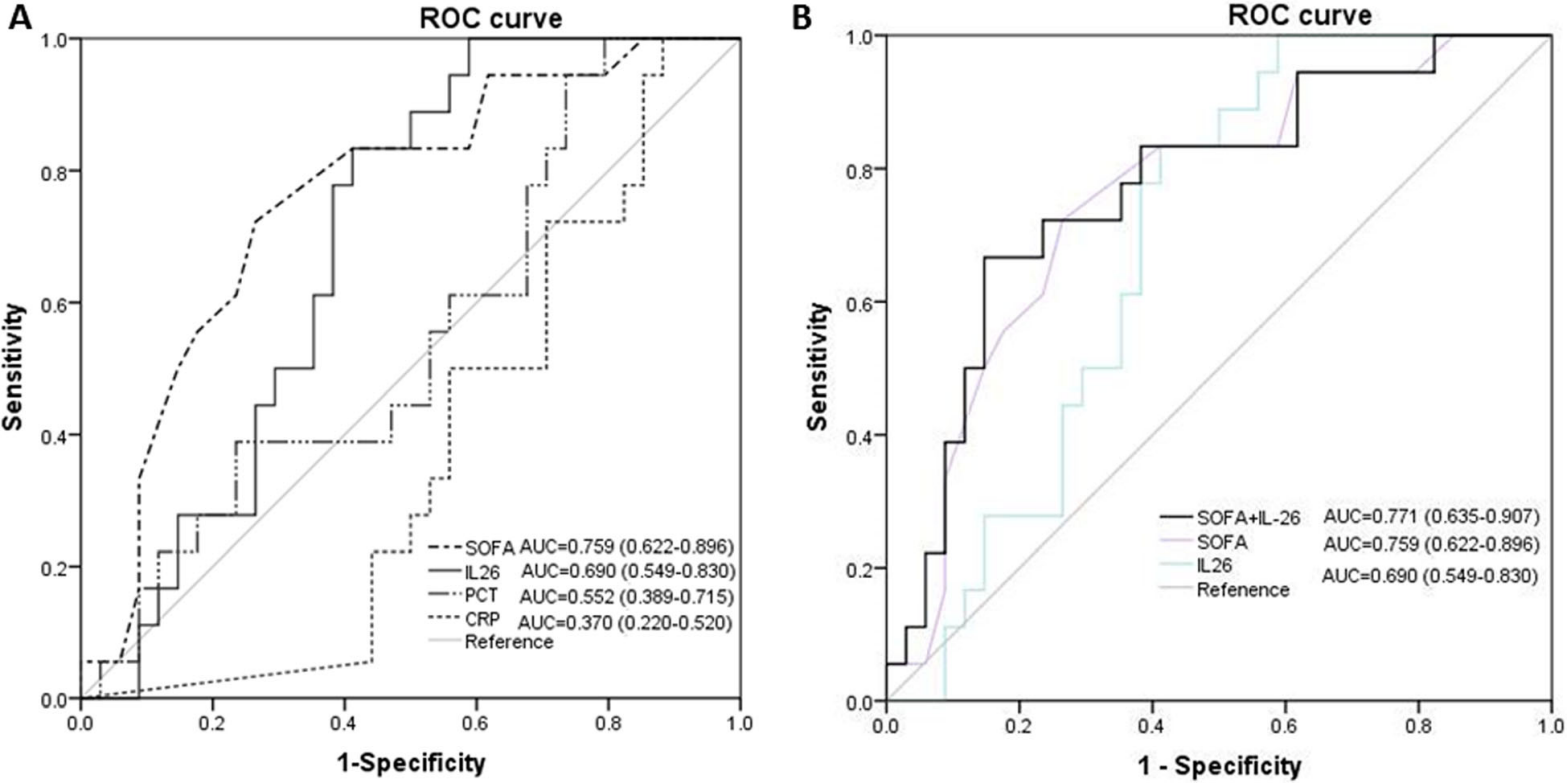
**a** Receiving operating characteristic (ROC) curve of IL-26, SOFA score, PCT, and CRP at admission for predicting 28-day mortality in septic patients. Area under the ROC curve, 0.690 (*p* = 0.036) for IL-26, 0.759 (*p* = 0.019) for SOFA score, 0.552 (*p* = 0.091) for PCT, and 0.370 (*p* = 0.265) for CRP. **b** Receiving operating characteristic (ROC) curve of IL-26 in combination with SOFA score for predicting 28-day mortality in septic patients. Area under ROC curve for the combination of IL-26 and SOFA score, 0.771 (*p* = 0.017)

The ROC curve of IL-26 for diagnosing sepsis is shown in Fig. 3. The AUC of IL-26 on day of ICU admission was 0.79 (*p* = 0.019, [95% CI] 0.69–0.89). We also investigated whether IL-26 was useful to predict 28-day mortality in septic patients; ROC analysis was conducted for prediction of 28-day mortality using serum IL-26 levels. Regarding the prediction of 28-day mortality (Fig. 4a), the area under the ROC curve for IL-26 on day of ICU admission was 0.69 (*p* = 0.036, [95% CI] 0.55–0.83), higher than the AUC for PCT (AUC = 0.55; [95% CI] 0.40–0.72, *p* = 0.091), and CRP (AUC = 0.37, [95% CI] 0.22–0.52, *p* = 0.26), but lower than the AUC for SOFA (AUC = 0.76; [95% CI] 0.62–0.90, *p* = 0.011). The AUC of IL-26 in combination with SOFA score was 0.77 ([95% CI] 0.64–0.91), which was more statistically significant compared with IL-26 alone (*p* = 0.016), and there was no difference for the combination of IL-26 and SOFA score when compared with SOFA score alone (Fig. 4b).

Using binary logistic regression analysis, both IL-26 (*B* = 0.001, OR = 1.001, *p* = 0.034) and SOFA score (*B* = 0.231, OR = 1.260, *p* = 0.015) on day of ICU admission were found to be independent predictors of 28-day mortality in the patients with sepsis, but PCT (*B* = 0.002, OR = 1.002, *p* = 0.521) was not (Table 1).

**Table 1:** Independent factors predicting 28-day mortality in septic patients

Variable	*B*	Standard error	Wald	Degrees of freedom	*P* value	Odds ratio	95% confidence interval
IL-26	0.001	0.001	4.908	1	0.034	1.001	0.999–1.004
SOFA	0.231	0.090	5.926	1	0.015	1.260	1.046–1.518
PCT	0.002	0.003	0.378	1	0.521	1.002	0.997–1.006

*PCT* procalcitonin, *SOFA* Sequential Organ Failure Assessment

### Administration of recombinant human IL-26 aggravated CLP-induced sepsis mortality

**Fig. 5:**
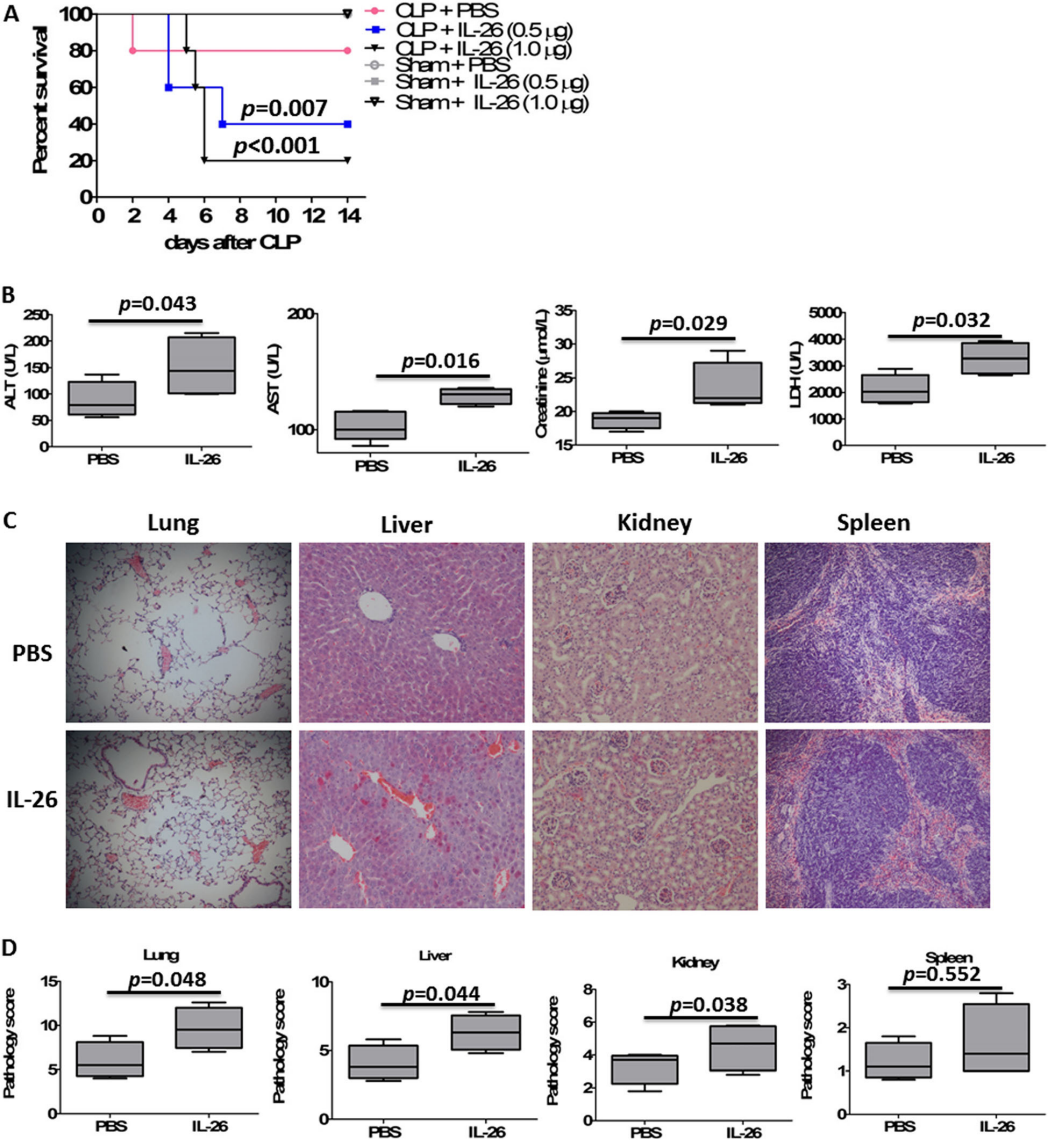
Effect of recombinant human IL-26 on cecal ligation and puncture (CLP)-induced inflammation and injury of vital organs and death. **a** Survival of CLP mice (*n* = 20 per group) after treatment with recombinant human IL-26 in the absence or presence of CLP-induced sepsis. Recombinant human IL-26 was injected intraperitoneally at 0.5–1.0 μg/injection immediately after CLP, and PBS was delivered in a similar fashion as control solutions. Comparison between groups was done by Kaplan–Meier analysis followed by log-rank tests. Results are representative of three independent experiments. **b** Serological markers of organ injury including alanine aminotransferase (ALT), aspartate aminotransferase (AST), lactate dehydrogenase (LDH), and creatinine in mice (*n* = 5 per group) treated with or without recombinant IL-26 (0.5 μg) at 24 h after CLP. Statistical difference was denoted by the horizontal bracket (Mann–Whitney *U* test). **c** Representative examples of hematoxylin and eosin (H&E)-stained lung, liver, spleen, and kidney tissues from mice (*n* = 5 per group) treated with or without recombinant human IL-26 (0.5 μg) at 24 h after CLP. **d** Histological scores for lung, liver, spleen, and kidney tissues from mice (*n* = 5 per group) treated with or without recombinant human IL-26 (0.5 μg) at 24 h after CLP. Statistical difference was denoted by the horizontal bracket (Mann–Whitney *U* test)

Given that mice do not express IL-26 and mice express both receptor chains that are necessary to form IL-26 receptor complexes [5], we therefore examine the effects of recombinant human IL-26 on the pathogenesis of sepsis in a CLP-induced polymicrobial sepsis model. In this study, we used our sublethal model of CLP that consistently caused 10–20% mortality and tested the effects of increasing doses (0.5–1.0 μg) of recombinant human IL-26 in mice with concomitant septic challenge. Supplementation with recombinant human IL-26 in the absence of sepsis had no effect on survival at doses up 1.0 μg in control mice with sham surgery. Injection of recombinant human IL-26 (0.5–1.0 μg) dramatically decreased septic mouse survival compared with the PBS-injected septic mice (Fig. 5a). The lowest dose of recombinant human IL-26 (0.5 μg) decreased sepsis survival from 80% (in PBS-treated septic mice) to 40%, and the highest dose of recombinant IL-26 (1.0 μg) decreased survival to 20%. Given these results, our subsequent experiments were performed in CLP mice, using recombinant human IL-26 (0.5 μg) immediately after CLP.

Exacerbated mortality of septic mice treated with recombinant IL-26 was closely associated with increased organ injury. Serum concentrations of ALT and AST, markers for hepatocellular injury; LDH, a marker for general cellular injury; and creatinine, a marker for renal failure, were significantly increased in septic mice treated with recombinant human IL-26 at 24 h after CLP (Fig. 5b). Lung, liver, and kidney damage was also confirmed by histological detection of alveolar hemorrhage, centrilobular necrosis, and tubular epithelial necrosis, respectively (Fig. 5c). In addition, administration of recombinant human IL-26 significantly increased the pathology scores for the lungs, livers, and kidneys after sepsis at 24 h after CLP (Fig. 5d).

### IL-26 selectively promoted neutrophil infiltration in septic mice

There were significantly greater numbers of leukocytes in peritoneal lavage fluid from mice treated with recombinant human IL-26 compared with mice treated with PBS control at 24 h after CLP (Additional file 1: Figure S1A). Notably, septic animals treated with recombinant human IL-26 had a significant increase in the numbers of neutrophils in peritoneal lavage fluids compared with those treated with PBS control (Additional file 1: Figure S1B). In contrast, we did not observe significant differences in the total numbers of macrophages and lymphocytes in peritoneal lavage fluids.

### IL-26 upregulated the production of pro-inflammatory cytokines and chemokines in septic mice

**Fig. 6:**
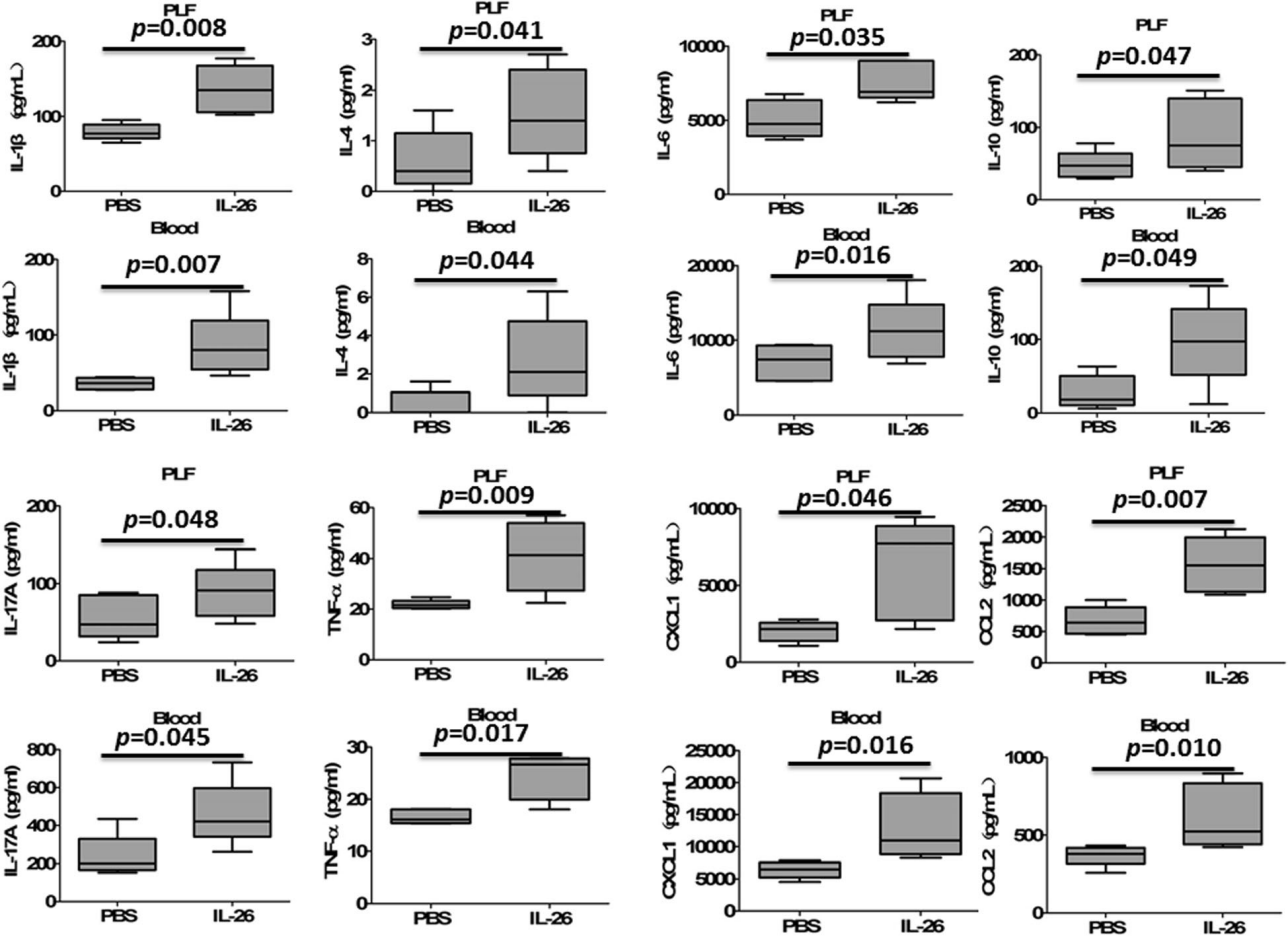
Administration with recombinant human IL-26 exaggerated the production of proinflammatory cytokines and chemokines in CLP-induced sepsis. Cytokine and chemokine concentrations in peritoneal lavage fluid (PLF) and blood from septic mice (*n* = 5) treated with or without recombinant human IL-26 (0.5 μg) were determined by ELISA at 24 h after CLP. Statistical difference was denoted by the horizontal bracket (Mann–Whitney *U* test)

Sepsis is characterized by dysregulated local and systemic inflammation [12]. The effect of recombinant human IL-26 on CLP-induced pro-inflammatory cytokines and chemokines in the peritoneal lavage fluid and blood was then evaluated. Treatment with recombinant human IL-26 enhanced the pro-inflammatory cytokine and chemokine response in the CLP model and resulted in a significant increase of IL-1β, IL-4, IL-6, IL-10, IL-17A, TNF-α, CXCL1, and CCL2 in the peritoneal lavage fluid and blood at 24 h after CLP (Fig. 6).

### IL-26 increased bacterial clearance in septic mice

**Fig. 7:**
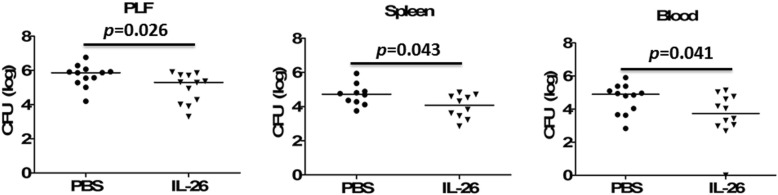
Administration with recombinant human IL-26 increased bacterial clearance in CLP-induced sepsis. Dilutions of peritoneal lavage fluid (PLF), spleen homogenates, and blood obtained from CLP mice (*n* = 5) treated with or without recombinant human IL-26 (0.5 μg) were cultured on blood agar plates, and the number of bacterial colonies was counted as colony-forming unit (CFU). Statistical difference was denoted by the horizontal bracket (Mann–Whitney *U* test)

To determine whether there would be any difference in the bacterial clearance, serial dilutions of peritoneal lavage fluids, spleen homogenates, and blood were cultured on blood agar plates in both aerobic and anaerobic conditions. The increased mortality rate in the mice treated with recombinant human IL-26 could not be explained by defective bacterial clearance, because local and systemic bacterial CFU levels were significantly decreased in the mice treated with recombinant IL-26 compared to mice treated with PBS control (Fig. 7). To evaluate phagocytic function more closely, peritoneal neutrophils and macrophages were harvested from naive mice. We observed a significant increase in neutrophil phagocytosis of *Escherichia coli* after the stimulation with recombinant human IL-26 (Additional file 2: Figure S2A), and there was a significant increase in bacterial killing from IL-26-stimulated neutrophils (Additional file 2: Figure S2B). However, recombinant human IL-26 did not affect macrophage-mediated bacterial phagocytosis (Additional file 3: Figure S3A) and killing (Additional file 3: Figure S3B).

## Discussion

Severe sepsis is characterized by systemic inflammation, organ injury, and a high rate of mortality [3, 12]. Cytokines play an important role in the pathogenesis of sepsis [13–15]. IL-26 is an emerging pro-inflammatory member of the IL-10 cytokine family with multifaceted actions in inflammatory responses [5, 16]. This study firstly assessed the production of serum IL-26 in patients with sepsis, resulting in three main findings. First, circulating IL-26 levels were significantly higher in septic patients on the day of ICU admission than in ICU controls and in healthy volunteers. Second, the serum IL-26 levels were related to the severity of sepsis, including parameters such as CRP, PCT, the length of ICU stay, and SOFA score. Third, a high serum IL-26 level on the day of ICU admission was associated with 28-day mortality in septic patients, and IL-26 was found to be an independent predictor of 28-day mortality in the patients with sepsis.

Previously, others have demonstrated elevated IL-26 levels in serum of human patients with various inflammatory disorders. In HCV infection, the serum levels of IL-26 were enhanced in chronically HCV-infected patients, mainly in those with severe liver inflammation [6]. In rheumatoid arthritis (RA), the serum concentrations of IL-26 were higher in RA patients than those of healthy subjects and dramatically elevated in RA synovial fluids compared to RA serums [17]. The current study extends these findings, not only by showing elevation of serum IL-26 in a well-defined cohort of septic patients, but also by providing information on correlations between serum IL-26 levels and sepsis-associated disease severity. Importantly, significantly higher serum levels of IL-26 were observed in septic nonsurvivors than survivors on the day of ICU admission. Although the AUC of IL-26 was lower than that of SOFA score in predicting 28-day mortality in septic patients, it was superior to PCT and CRP for predicting 28-day mortality in septic patients. In the logistic regression analysis, both IL-26 and SOFA score were found to be independent predictors of 28-day mortality in septic patients, while PCT was not. Our results suggest that elevated serum level of IL-26 was associated with increased mortality in septic patients, and IL-26 may add potential value for risk stratification and evaluation of prognosis in septic patients.

To further investigate the role of IL-26 in the pathogenesis of sepsis, we established a clinically relevant murine model of sepsis induced by CLP. Since the murine genome does not appear to contain a paralog of the IL-26 gene, we could not use IL-26-knockout mice to examine the contribution of IL-26 to host immune response during sepsis. However, mice do express IL-26 receptor complexes that human IL-26 protein can bind and signal through [5]. Consequently, we tested the effect of human recombinant human IL-26 in mice with concomitant septic challenge. As described previously that IL-26 is a proinflammatory cytokine with antimicrobial functions [18, 19], we found that recombinant human IL-26 enhanced septic mice to clear bacteria. However, this antibacterial defense induced by recombinant human IL-26 could not increase the survival of septic mice. On the contrary, administration with recombinant human IL-26 was detrimental in murine sepsis, and it aggravated CLP-induced sepsis mortality, which was associated with systemic inflammatory reactions (increased proinflammatory cytokines and chemokines) and multiple organ injury (liver, lung, and kidney). Interestingly, accumulation of neutrophils was predominantly augmented in the peritoneal cavity of septic mice treated with recombinant human IL-26, which could contribute to an effective local antibacterial defense. On the other hand, excessive neutrophils also injured the host. Likewise, a recent study has also shown that recombinant human IL-26 potentiated neutrophil migration toward the bacterial compound fMLP and the archetype chemokine CXCL8 [20].

A variety of studies have shown that IL-26 could enhance inflammatory response in RA [17], inflammatory bowel disease (IBD) [7], or HCV infection [6]. During sepsis, inflammatory response may act as double-edged swords, fighting pathogens on the one hand, but potentially causing tissue damage on the other hand [21, 22]. Here we demonstrated that IL-26 was an important sepsis-related proinflammatory cytokine, which contributed to inflammatory response, organ injury, and mortality in murine sepsis. The supplementation of recombinant human IL-26 was detrimental to the survival of mice, accompanied by aberrant inflammation and multiple organ damage, despite a more effective bacterial clearance and a reduced dissemination of bacteria to distant organs. Taking into consideration the beneficial effects of IL-26 on bacterial clearance, we can speculate that IL-26 elevation during sepsis is an immunoregulatory mechanism that is initiated by the host to combat bacterial infection. However, excessive IL-26 amounts could exaggerate inflammatory response (increase of neutrophil infiltration and elevation of inflammatory cytokines/chemokines including IL-1β, IL-4, IL-6, IL-10, IL-17A, TNF-α, CXCL1, and CCL2), resulting in aberrant inflammation, which can cause multiple organ damage, shock, and death. Therefore, we should point that the net effect of endogenous IL-26 on the outcome of human sepsis is determined by the balance between its local effects (facilitating the clearance of microorganisms) and its systemic effects (exaggerating inflammation). Similarly, a recent study has shown that thymic stromal lymphopoietin (TSLP) contributed to survival and reduced morbidity by limiting inflammation, despite the impaired bacteria clearance, after CLP-induced polymicrobial sepsis [23]. Taken together, these data further confirm the importance of inflammation in sepsis-induced lethality caused by bacterial infection. The inflammatory response induced by the bacterial infection, which was regulated by the host factors, rather than the bacteria itself, determined the outcome during sepsis.

Some limitations of the present study should be considered. First, the small sample size in this study did not allow in-depth analysis of the relationships between IL-26 levels and disease characteristics or severity as well as mortality, which means that the clinical values of IL-26 for diagnosing sepsis and for predicting mortality require to be confirmed in a larger ideally multicentric study including important cohorts of septic patients. Second, although we have included healthy controls and non-sepsis critical illness controls in this study, and this method has been widely accepted and used [24–26], other groups of patient controls should be included, such as systemic inflammatory response syndrome (SIRS) patients without infection, or patients who had an increase in SOFA of 2 or more without infection in the definition of Sepsis 3. Third, although the prediction capacity of IL-26 seemed to outperform PCT and CRP in predicting 28-day mortality in septic patients, the AUC of IL-26 was lower than that of SOFA score. However, the combination of IL-26 with SOFA score could enhance the accuracy of predicting 28-day mortality in septic patients. Therefore, IL-26 had some, albeit limited, incremental prognostic value. Future studies need to explore whether the combination of IL-26 with other biomarkers might provide additional value in predicting mortality in septic patients. Fourth, blood sample was obtained on the day of ICU admission. This does not exclude, however, that sepsis was already present prior to ICU admission, although given the severity of sepsis, it seems impossible that this would have occurred in most patients and/or for prolonged time periods. Fifth, the kinetics of IL-26 secretion, the cellular source of IL-26, and the factors regulating IL-26 production in human sepsis remain unknown. Finally, the relevance of our murine findings regarding the detrimental contribution of recombinant human IL-26 to the regulation of inflammation in human sepsis still requires investigation, and we acknowledge that this can be further established in mice by knocking out IL-26 receptors and in humans using anti-IL-26 neutralizing antibodies or other specific IL-26 blockers.

## Conclusions

This study firstly showed that the serum IL-26 level was significantly elevated in human sepsis on day of ICU admission, which correlated with disease severity and 28-day mortality. Furthermore, our data demonstrated a previously unrecognized role of IL-26 in increasing lethality during experimental sepsis despite promoting antibacterial host responses. Further investigations are warranted to investigate whether IL-26 treatment or blockade in sepsis has the potential to influence the outcome of the disease in humans.

## Additional files

Additional file 1: **Figure S1.** Treatment with recombinant human IL-26 enhanced neutrophil infiltration during CLP-induced sepsis. (A) Cytospin centrifugation was performed for Diff-Quik staining (× 10) to assess cell counts in PLF from septic mice (*n* = 5) treated with or without recombinant human IL-26 (0.5 μg) at 24 h after CLP. (B) Number of leukocytes in peritoneal lavage fluid (PLF) from mice (*n* = 5) treated with or without recombinant human IL-26 (0.5 μg) at 24 h after CLP. Statistical difference was denoted by the horizontal bracket (Mann–Whitney *U* test). (TIF 466 kb)

Additional file 2: **Figure S2.** Treatment with recombinant human IL-26 enhanced bacterial phagocytosis and killing by neutrophils. (A) Peritoneal mouse neutrophils were pretreated with or without recombinant human IL-26 (100 ng/ml) for 6 h and then infected with FITC-labeled *E. coli* for 30 min. Arrows indicate engulfed bacteria (as determined by overlay of green bacteria) by neutrophils. A representative experiment was shown. Identical results were obtained with cells from 5 independent experiments. Data were expressed as mean ± SD and were analyzed using the Student’s t-test. (B) Peritoneal mouse neutrophils were pretreated with recombinant human IL-26 (100 ng/mL) for 6 h and then infected with live *E. coli* (multiplicity of infection, 100). Extracellular bacteria were then removed by washing with tobramycin. Cells were lysed, and live intracellular bacteria levels were determined by culture for evaluation of intracellular killing (t = 1 h). Data were expressed as mean ± SD from 5 independent experiments and were analyzed using the Student’s *t*-test. Statistical difference was denoted by the horizontal bracket. (TIF 290 kb)

Additional file 3: **Figure S3.** Effect of recombinant human IL-26 on bacterial phagocytosis and killing by macrophages. (A) Peritoneal macrophages were stimulated with recombinant human IL-26 (100 ng/mL) for 12 h and then challenged with FITC-labeled *E. coli* for 30 min at 37 °C. Arrows indicate engulfed bacteria (as determined by overlay of green bacteria) by macrophages. A representative experiment is shown. Identical results were obtained with cells from 5 independent experiments. Data were expressed as mean ± SD and were analyzed using the Student’s t-test. (B) Peritoneal mouse macrophages were pretreated with recombinant human IL-26 (100 ng/mL) for 6 h and then infected with live *E. coli* (multiplicity of infection, 10). Extracellular bacteria were then removed by washing with tobramycin. Cells were lysed, and live intracellular bacteria levels were determined by culture for evaluation of intracellular killing (t = 2 h). Data were expressed as mean ± SD from 5 independent experiments and were analyzed using the Student’s *t*-test. (TIF 221 kb)

Additional file 4: **Table S1.** Characteristics of septic patients, ICU controls and healthy controls. (DOCX 25 kb)

Additional file 5: **Table S2.** Characteristics of septic survivor and non-survivor. (DOCX 24 kb)

## Data Availability

All data generated or analyzed during this study are included in this published article.

## Abbreviations

ALT: Alanine transaminase
AST: Aspartate transaminase
AUC: Area under the curve
CI: Confidence interval
CFU: Colony-forming unit
CLP: Cecal ligation and puncture
CRP: C-reaction protein
IL-26: Interleukin-26
LDH: Lactate dehydrogenase
PCT: Procalcitonin
PLF: Peritoneal lavage fluid
ROC: Receiver-operating characteristic
SD: Standard deviation
SOFA: Sequential [Sepsis-related] Organ Failure Assessment
WBC: White blood cells
