# Supplementary figures and images for: Interleukin-26 is overexpressed in human sepsis and contributes to inflammation, organ injury, and mortality in murine sepsis
Source: Crit Care. 2019 Aug 29;23:290. doi: 10.1186/s13054-019-2574-7 (PMC6716900; doi:10.1186/s13054-019-2574-7)

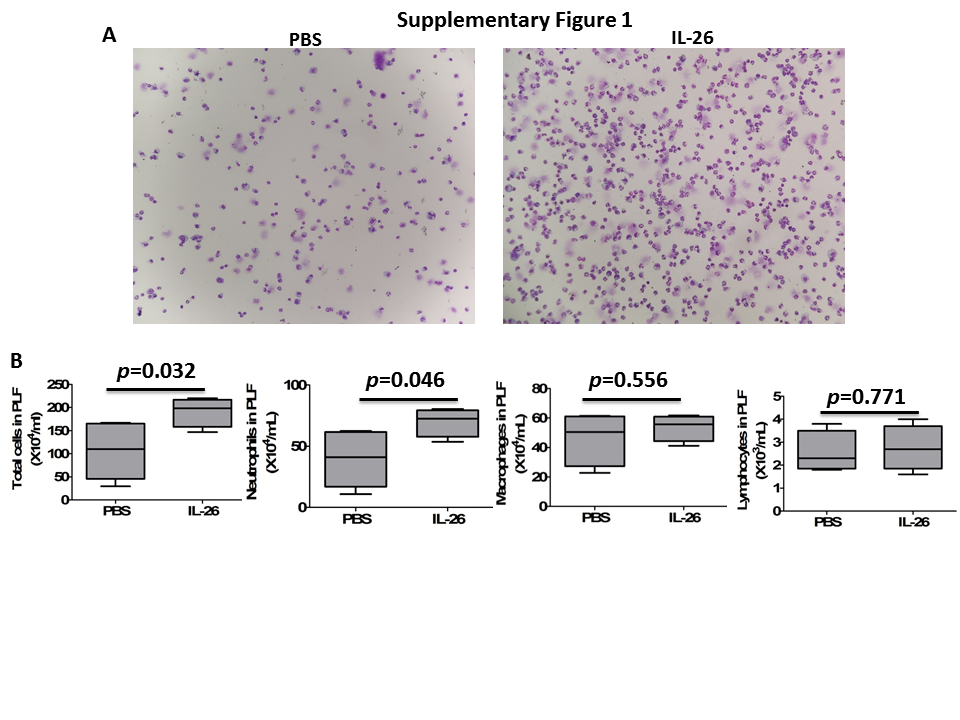

Supplement: Supplementary file 1 — Figure S1. Treatment with recombinant human IL-26 enhanced neutrophil infiltration during CLP-induced sepsis. (A) Cytospin centrifugation was performed for Diff-Quik staining (× 10) to assess cell counts in PLF from septic mice (n = 5) treated with or without recombinant human IL-26 (0.5 μg) at 24 h after CLP. (B) Number of leukocytes in peritoneal lavage fluid (PLF) from mice (n = 5) treated with or without recombinant human IL-26 (0.5 μg) at 24 h after CLP. Statistical difference was denoted by the horizontal bracket (Mann–Whitney U test). (TIF 466 kb) [file 13054_2019_2574_MOESM1_ESM.tif]

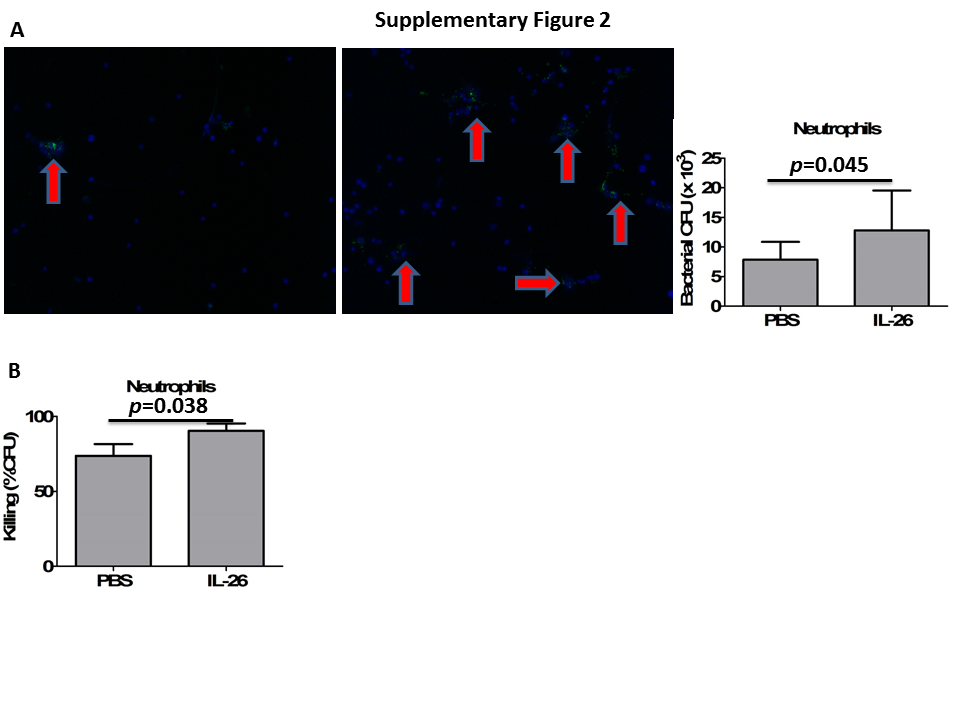

Supplement: Supplementary file 2 — Figure S2. Treatment with recombinant human IL-26 enhanced bacterial phagocytosis and killing by neutrophils. (A) Peritoneal mouse neutrophils were pretreated with or without recombinant human IL-26 (100 ng/ml) for 6 h and then infected with FITC-labeled E. coli for 30 min. Arrows indicate engulfed bacteria (as determined by overlay of green bacteria) by neutrophils. A representative experiment was shown. Identical results were obtained with cells from 5 independent experiments. Data were expressed as mean ± SD and were analyzed using the Student’s t-test. (B) Peritoneal mouse neutrophils were pretreated with recombinant human IL-26 (100 ng/mL) for 6 h and then infected with live E. coli (multiplicity of infection, 100). Extracellular bacteria were then removed by washing with tobramycin. Cells were lysed, and live intracellular bacteria levels were determined by culture for evaluation of intracellular killing (t = 1 h). Data were expressed as mean ± SD from 5 independent experiments and were analyzed using the Student’s t-test. Statistical difference was denoted by the horizontal bracket. (TIF 290 kb) [file 13054_2019_2574_MOESM2_ESM.tif]

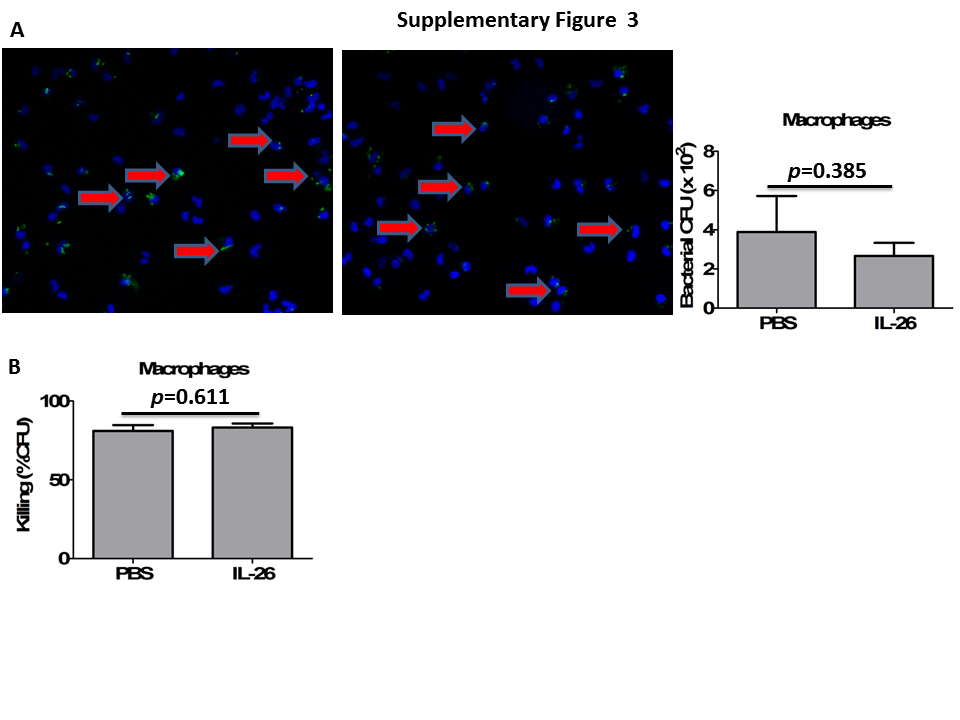

Supplement: Supplementary file 3 — Figure S3. Effect of recombinant human IL-26 on bacterial phagocytosis and killing by macrophages. (A) Peritoneal macrophages were stimulated with recombinant human IL-26 (100 ng/mL) for 12 h and then challenged with FITC-labeled E. coli for 30 min at 37 °C. Arrows indicate engulfed bacteria (as determined by overlay of green bacteria) by macrophages. A representative experiment is shown. Identical results were obtained with cells from 5 independent experiments. Data were expressed as mean ± SD and were analyzed using the Student’s t-test. (B) Peritoneal mouse macrophages were pretreated with recombinant human IL-26 (100 ng/mL) for 6 h and then infected with live E. coli (multiplicity of infection, 10). Extracellular bacteria were then removed by washing with tobramycin. Cells were lysed, and live intracellular bacteria levels were determined by culture for evaluation of intracellular killing (t = 2 h). Data were expressed as mean ± SD from 5 independent experiments and were analyzed using the Student’s t-test. (TIF 221 kb) [file 13054_2019_2574_MOESM3_ESM.tif]
