# Supplementary material for: Interleukin-26 is overexpressed in human sepsis and contributes to inflammation, organ injury, and mortality in murine sepsis
Source: Crit Care. 2019 Aug 29;23:290. doi: 10.1186/s13054-019-2574-7 (PMC6716900; doi:10.1186/s13054-019-2574-7)
Supplement: Supplementary file 4 — Table S1. Characteristics of septic patients, ICU controls and healthy controls. (DOCX 25 kb) [file 13054_2019_2574_MOESM4_ESM.docx]

**Supplementary Table 1 Characteristics of septic patients, ICU controls and healthy controls**

Characteristics Sepsis patients (n=52) ICU controls (n=18) Healthy controls (n=30)

Male sex 35 12 21

Age, years 59 (47 – 81) 52 (45 – 69) 55 (44 – 76)

WBC, 10^9^/L 14 (9 – 22) 7 (4 – 9) 6 (4 – 9)

CRP, mg/L 148.8 (26.5 – 200.0) 4.6 (0 – 13.9) NA

PCT, ng/ml 18.6 (0.3 – 200.0) 0.1 (0.0 – 0.5) NA

Infection site, no. of patients

Respiratory 22 NA NA

Abdominal 15 NA NA

Vascular 6 NA NA

Urinary 6 NA NA

Other 3 NA NA

Bacteremia 30 NA NA

Isolates, no. of patients

Gram-positive 15 NA NA

Gram-negative 27 NA NA

Polybacterial 7 NA NA

Fungi 3 NA NA

SOFA score 8.0 (2.0-19.0) 1.0 (0.3-2.3) NA

ICU stay, days 10 (6-16) 3 (1-6) NA

Died/survived 18/34 0/18 NA

NOTE. Data are expressed as median (interquartile range) unless otherwise indicated. CRP: C-reaction protein; ICU: intensive care unit; PCT: procalcitonin; SOFA: sequential organ failure assessment; WBC: white blood cells; NA: not applicable.
