# Supplementary material for: Interleukin-26 is overexpressed in human sepsis and contributes to inflammation, organ injury, and mortality in murine sepsis
Source: Crit Care. 2019 Aug 29;23:290. doi: 10.1186/s13054-019-2574-7 (PMC6716900; doi:10.1186/s13054-019-2574-7)
Supplement: Supplementary file 5 — Table S2. Characteristics of septic survivor and non-survivor. (DOCX 24 kb) [file 13054_2019_2574_MOESM5_ESM.docx]

**Supplementary Table 2 Characteristics of septic survivor and non-survivor**

Characteristics Survivors (n=34) Non-survivors (n=18)

Male sex 23 12

Age, years 57 (49 – 81) 60 (47 – 77)

WBC, 10^9^/L 14 (10 – 22) 15 (9 – 19)

Lactates, mmol/L 2.1 (1.2 – 4.3) 3.3 (1.8 – 6.2)*

SOFA score 7.0 (2.0 – 12.0) 10.0 (6.0 – 19.0)**

ICU stay, days 9 (3 – 16) 10 (6 – 15)

Any hydrocortisone use^#^ 22 18

NOTE. Data are expressed as median (interquartile range) unless otherwise indicated. SOFA: sequential organ failure assessment; ICU: intensive care unit; WBC: white blood cells

^#^Use of hydrocortisone or its equivalent (hydrocortisone dose = 4 x prednisolone dose, 5 x methylprednisolone dose, 25 x dexamethasone dose).

**p*=0.025, ***p*=0.007 when compared with survivors (Mann–Whitney *U* test).
